# A novel *gyrB* gene mutation in fluoroquinolone resistant clinical isolates of *Mycobacterium tuberculosis*

**DOI:** 10.1186/1471-2334-14-S3-O14

**Published:** 2014-05-27

**Authors:** Pooja Singh, Amita Jain, Pratima Dixit, Shantanu Prakash, Indu Jaiswal, Vimala Venkatesh, Mastan Singh

**Affiliations:** 1 Department of Microbiology, King George’s Medical University, Lucknow, UP, India

## Introduction

Fluoroquinolone (FQ) resistance in *Mycobacterium tuberculosis* can be conferred by mutations in *gyrA* or *gyrB* gene. Recent studies suggest that amino acid substitutions in *gyrB* gene may also play a crucial role in resistance, but genetic studies of these mutations in *M. tuberculosis* are lacking.

## Methods

A total of 100 ofloxacin resistant (OFX r) and 100 OFX sensitive (OFX s) isolates of *M.tuberculosis* isolates were consecutively selected from routine TB laboratory during 2012-2013. All the isolates were screened for phenotypic OFX r (>2µg/ml) by 1% proportion method and tested for minimal inhibitory concentration by absolute concentration method. Quinolone resistance determining region (QRDR) of *gyrA* and *gyrB* genes of 320bp and 428bp respectively were amplified, sequenced and compared with *M.tuberculosis* H37Rv.

## Results

Mutations in the *gyrB* gene were observed in 5 of the 100 OFX r isolates. The single nucleotide mutation sites were in codons 538, 500, 539 (in two isolates) and 592. In one isolate, a substitution at codon 592 (Pro592Ser) was found as novel mutation outside QRDR region of *gyrB* gene. Accession nos. of these isolates include; KF509920-KF509922, KC880086 & KC880101. All the isolates showing mutations in *gyrB* gene also had mutations in *gyrA* gene. Mutations in *gyrA* gene were observed in 79% OFX r isolates. No mutation was observed in *gyrB* gene of OFX s isolates.

## Conclusion

No OFX r isolates had shown mutation in the *gyrB* gene in the absence of *gyrA* gene mutation. The role of the *gyrB* gene mutation in conferring resistance to OFX in *M.tuberculosis* needs to be studied further.

